# Supplementary material for: Pyruvate Dehydrogenase Kinase 1 inhibition mediated oxidative phosphorylation enhancement in cartilage promotes osteoarthritis progression
Source: BMC Musculoskelet Disord. 2023 Jul 20;24:597. doi: 10.1186/s12891-023-06585-6 (PMC10357736; doi:10.1186/s12891-023-06585-6)
Supplement: Supplementary file 1 — Additional file 1. [file 12891_2023_6585_MOESM1_ESM.zip › KEGG.Gsea.1653622432464/KEGG_OXIDATIVE_PHOSPHORYLATION.html]

Details for gene set KEGG\_OXIDATIVE\_PHOSPHORYLATION[GSEA]

|  || Dataset | 55457\_collapsed\_to\_symbols.cls.cls #OA\_versus\_normal.cls.cls #OA\_versus\_normal\_repos |
| Phenotype | cls.cls#OA\_versus\_normal\_repos |
| Upregulated in class | OA |
| GeneSet | KEGG\_OXIDATIVE\_PHOSPHORYLATION |
| Enrichment Score (ES) | 0.6313611 |
| Normalized Enrichment Score (NES) | 2.9534543 |
| Nominal p-value | 0.0 |
| FDR q-value | 0.0 |
| FWER p-Value | 0.0 |
Table: GSEA Results Summary

  

Fig 1: Enrichment plot: KEGG\_OXIDATIVE\_PHOSPHORYLATION      
 Profile of the Running ES Score & Positions of GeneSet Members on the Rank Ordered List

  

| SYMBOL | TITLE | RANK IN GENE LIST | RANK METRIC SCORE | RUNNING ES | CORE ENRICHMENT || 1 | ATP6V1C1 | ATPase H+ transporting V1 subunit C1 [Source:HGNC Symbol;Acc:HGNC:856] | 17 | 1.125 | 0.0255 | Yes |
| 2 | UQCR10 | "ubiquinol-cytochrome c reductase, complex III subunit X [Source:HGNC Symbol;Acc:HGNC:30863]" | 53 | 0.987 | 0.0464 | Yes |
| 3 | COX17 | cytochrome c oxidase copper chaperone COX17 [Source:HGNC Symbol;Acc:HGNC:2264] | 64 | 0.966 | 0.0687 | Yes |
| 4 | NDUFA8 | NADH:ubiquinone oxidoreductase subunit A8 [Source:HGNC Symbol;Acc:HGNC:7692] | 67 | 0.961 | 0.0915 | Yes |
| 5 | ATP6V0D1 | ATPase H+ transporting V0 subunit d1 [Source:HGNC Symbol;Acc:HGNC:13724] | 135 | 0.872 | 0.1070 | Yes |
| 6 | NDUFA4L2 | NDUFA4 mitochondrial complex associated like 2 [Source:HGNC Symbol;Acc:HGNC:29836] | 150 | 0.861 | 0.1265 | Yes |
| 7 | NDUFS3 | NADH:ubiquinone oxidoreductase core subunit S3 [Source:HGNC Symbol;Acc:HGNC:7710] | 177 | 0.836 | 0.1444 | Yes |
| 8 | NDUFA1 | NADH:ubiquinone oxidoreductase subunit A1 [Source:HGNC Symbol;Acc:HGNC:7683] | 194 | 0.826 | 0.1629 | Yes |
| 9 | NDUFA10 | NADH:ubiquinone oxidoreductase subunit A10 [Source:HGNC Symbol;Acc:HGNC:7684] | 243 | 0.788 | 0.1780 | Yes |
| 10 | NDUFB6 | NADH:ubiquinone oxidoreductase subunit B6 [Source:HGNC Symbol;Acc:HGNC:7701] | 262 | 0.779 | 0.1952 | Yes |
| 11 | ATP6V1E1 | ATPase H+ transporting V1 subunit E1 [Source:HGNC Symbol;Acc:HGNC:857] | 287 | 0.758 | 0.2114 | Yes |
| 12 | ATP6V0B | ATPase H+ transporting V0 subunit b [Source:HGNC Symbol;Acc:HGNC:861] | 382 | 0.713 | 0.2211 | Yes |
| 13 | ATP5MC1 | ATP synthase membrane subunit c locus 1 [Source:HGNC Symbol;Acc:HGNC:841] | 465 | 0.674 | 0.2308 | Yes |
| 14 | UQCRC1 | ubiquinol-cytochrome c reductase core protein 1 [Source:HGNC Symbol;Acc:HGNC:12585] | 473 | 0.672 | 0.2463 | Yes |
| 15 | NDUFB3 | NADH:ubiquinone oxidoreductase subunit B3 [Source:HGNC Symbol;Acc:HGNC:7698] | 483 | 0.667 | 0.2615 | Yes |
| 16 | LHPP | phospholysine phosphohistidine inorganic pyrophosphate phosphatase [Source:HGNC Symbol;Acc:HGNC:30042] | 491 | 0.662 | 0.2768 | Yes |
| 17 | ATP6V1G1 | ATPase H+ transporting V1 subunit G1 [Source:HGNC Symbol;Acc:HGNC:864] | 515 | 0.655 | 0.2907 | Yes |
| 18 | NDUFS4 | NADH:ubiquinone oxidoreductase subunit S4 [Source:HGNC Symbol;Acc:HGNC:7711] | 531 | 0.647 | 0.3049 | Yes |
| 19 | ATP6V0E2 | ATPase H+ transporting V0 subunit e2 [Source:HGNC Symbol;Acc:HGNC:21723] | 532 | 0.647 | 0.3204 | Yes |
| 20 | NDUFA3 | NADH:ubiquinone oxidoreductase subunit A3 [Source:HGNC Symbol;Acc:HGNC:7686] | 550 | 0.641 | 0.3344 | Yes |
| 21 | ATP5PD | ATP synthase peripheral stalk subunit d [Source:HGNC Symbol;Acc:HGNC:845] | 564 | 0.635 | 0.3485 | Yes |
| 22 | COX5B | cytochrome c oxidase subunit 5B [Source:HGNC Symbol;Acc:HGNC:2269] | 585 | 0.628 | 0.3620 | Yes |
| 23 | NDUFA4 | NDUFA4 mitochondrial complex associated [Source:HGNC Symbol;Acc:HGNC:7687] | 624 | 0.613 | 0.3737 | Yes |
| 24 | COX6A1 | cytochrome c oxidase subunit 6A1 [Source:HGNC Symbol;Acc:HGNC:2277] | 653 | 0.605 | 0.3859 | Yes |
| 25 | ATP6V1D | ATPase H+ transporting V1 subunit D [Source:HGNC Symbol;Acc:HGNC:13527] | 750 | 0.581 | 0.3923 | Yes |
| 26 | ATP5PO | ATP synthase peripheral stalk subunit OSCP [Source:HGNC Symbol;Acc:HGNC:850] | 840 | 0.558 | 0.3987 | Yes |
| 27 | NDUFA2 | NADH:ubiquinone oxidoreductase subunit A2 [Source:HGNC Symbol;Acc:HGNC:7685] | 878 | 0.549 | 0.4089 | Yes |
| 28 | NDUFB5 | NADH:ubiquinone oxidoreductase subunit B5 [Source:HGNC Symbol;Acc:HGNC:7700] | 902 | 0.542 | 0.4201 | Yes |
| 29 | NDUFC2 | NADH:ubiquinone oxidoreductase subunit C2 [Source:HGNC Symbol;Acc:HGNC:7706] | 929 | 0.532 | 0.4307 | Yes |
| 30 | NDUFA9 | NADH:ubiquinone oxidoreductase subunit A9 [Source:HGNC Symbol;Acc:HGNC:7693] | 943 | 0.529 | 0.4424 | Yes |
| 31 | NDUFB4 | NADH:ubiquinone oxidoreductase subunit B4 [Source:HGNC Symbol;Acc:HGNC:7699] | 968 | 0.525 | 0.4530 | Yes |
| 32 | UQCRQ | ubiquinol-cytochrome c reductase complex III subunit VII [Source:HGNC Symbol;Acc:HGNC:29594] | 986 | 0.521 | 0.4641 | Yes |
| 33 | NDUFB7 | NADH:ubiquinone oxidoreductase subunit B7 [Source:HGNC Symbol;Acc:HGNC:7702] | 1033 | 0.510 | 0.4727 | Yes |
| 34 | NDUFB1 | NADH:ubiquinone oxidoreductase subunit B1 [Source:HGNC Symbol;Acc:HGNC:7695] | 1055 | 0.506 | 0.4832 | Yes |
| 35 | NDUFS7 | NADH:ubiquinone oxidoreductase core subunit S7 [Source:HGNC Symbol;Acc:HGNC:7714] | 1071 | 0.501 | 0.4940 | Yes |
| 36 | NDUFC1 | NADH:ubiquinone oxidoreductase subunit C1 [Source:HGNC Symbol;Acc:HGNC:7705] | 1091 | 0.499 | 0.5044 | Yes |
| 37 | NDUFA7 | NADH:ubiquinone oxidoreductase subunit A7 [Source:HGNC Symbol;Acc:HGNC:7691] | 1097 | 0.496 | 0.5159 | Yes |
| 38 | COX8A | cytochrome c oxidase subunit 8A [Source:HGNC Symbol;Acc:HGNC:2294] | 1105 | 0.494 | 0.5271 | Yes |
| 39 | ATP6V0E1 | ATPase H+ transporting V0 subunit e1 [Source:HGNC Symbol;Acc:HGNC:863] | 1207 | 0.474 | 0.5306 | Yes |
| 40 | NDUFS8 | NADH:ubiquinone oxidoreductase core subunit S8 [Source:HGNC Symbol;Acc:HGNC:7715] | 1208 | 0.474 | 0.5419 | Yes |
| 41 | NDUFS1 | NADH:ubiquinone oxidoreductase core subunit S1 [Source:HGNC Symbol;Acc:HGNC:7707] | 1229 | 0.471 | 0.5516 | Yes |
| 42 | ATP5PB | ATP synthase peripheral stalk-membrane subunit b [Source:HGNC Symbol;Acc:HGNC:840] | 1293 | 0.459 | 0.5576 | Yes |
| 43 | ATP6V1H | ATPase H+ transporting V1 subunit H [Source:HGNC Symbol;Acc:HGNC:18303] | 1304 | 0.457 | 0.5678 | Yes |
| 44 | ATP5MF | ATP synthase membrane subunit f [Source:HGNC Symbol;Acc:HGNC:848] | 1330 | 0.453 | 0.5766 | Yes |
| 45 | NDUFS6 | NADH:ubiquinone oxidoreductase subunit S6 [Source:HGNC Symbol;Acc:HGNC:7713] | 1417 | 0.441 | 0.5804 | Yes |
| 46 | COX11 | cytochrome c oxidase copper chaperone COX11 [Source:HGNC Symbol;Acc:HGNC:2261] | 1622 | 0.410 | 0.5743 | Yes |
| 47 | NDUFV2 | NADH:ubiquinone oxidoreductase core subunit V2 [Source:HGNC Symbol;Acc:HGNC:7717] | 1642 | 0.407 | 0.5825 | Yes |
| 48 | NDUFV1 | NADH:ubiquinone oxidoreductase core subunit V1 [Source:HGNC Symbol;Acc:HGNC:7716] | 1663 | 0.404 | 0.5906 | Yes |
| 49 | SDHB | succinate dehydrogenase complex iron sulfur subunit B [Source:HGNC Symbol;Acc:HGNC:10681] | 1818 | 0.382 | 0.5877 | Yes |
| 50 | ATP6AP1 | ATPase H+ transporting accessory protein 1 [Source:HGNC Symbol;Acc:HGNC:868] | 1866 | 0.377 | 0.5930 | Yes |
| 51 | COX7A1 | cytochrome c oxidase subunit 7A1 [Source:HGNC Symbol;Acc:HGNC:2287] | 2045 | 0.356 | 0.5876 | Yes |
| 52 | COX4I1 | cytochrome c oxidase subunit 4I1 [Source:HGNC Symbol;Acc:HGNC:2265] | 2167 | 0.342 | 0.5863 | Yes |
| 53 | ATP5F1C | ATP synthase F1 subunit gamma [Source:HGNC Symbol;Acc:HGNC:833] | 2190 | 0.339 | 0.5927 | Yes |
| 54 | SDHA | succinate dehydrogenase complex flavoprotein subunit A [Source:HGNC Symbol;Acc:HGNC:10680] | 2231 | 0.335 | 0.5976 | Yes |
| 55 | NDUFS5 | NADH:ubiquinone oxidoreductase subunit S5 [Source:HGNC Symbol;Acc:HGNC:7712] | 2267 | 0.330 | 0.6028 | Yes |
| 56 | ATP12A | ATPase H+/K+ transporting non-gastric alpha2 subunit [Source:HGNC Symbol;Acc:HGNC:13816] | 2330 | 0.323 | 0.6056 | Yes |
| 57 | ATP5MC2 | ATP synthase membrane subunit c locus 2 [Source:HGNC Symbol;Acc:HGNC:842] | 2371 | 0.318 | 0.6101 | Yes |
| 58 | NDUFB2 | NADH:ubiquinone oxidoreductase subunit B2 [Source:HGNC Symbol;Acc:HGNC:7697] | 2460 | 0.307 | 0.6106 | Yes |
| 59 | COX7A2L | cytochrome c oxidase subunit 7A2 like [Source:HGNC Symbol;Acc:HGNC:2289] | 2517 | 0.302 | 0.6134 | Yes |
| 60 | NDUFB8 | NADH:ubiquinone oxidoreductase subunit B8 [Source:HGNC Symbol;Acc:HGNC:7703] | 2623 | 0.288 | 0.6121 | Yes |
| 61 | ATP5MC3 | ATP synthase membrane subunit c locus 3 [Source:HGNC Symbol;Acc:HGNC:843] | 2643 | 0.286 | 0.6174 | Yes |
| 62 | ATP5F1D | ATP synthase F1 subunit delta [Source:HGNC Symbol;Acc:HGNC:837] | 2701 | 0.279 | 0.6196 | Yes |
| 63 | ATP6V0C | ATPase H+ transporting V0 subunit c [Source:HGNC Symbol;Acc:HGNC:855] | 2790 | 0.271 | 0.6192 | Yes |
| 64 | COX6B1 | cytochrome c oxidase subunit 6B1 [Source:HGNC Symbol;Acc:HGNC:2280] | 2915 | 0.259 | 0.6157 | Yes |
| 65 | ATP5MG | ATP synthase membrane subunit g [Source:HGNC Symbol;Acc:HGNC:14247] | 2941 | 0.256 | 0.6199 | Yes |
| 66 | ATP5F1B | ATP synthase F1 subunit beta [Source:HGNC Symbol;Acc:HGNC:830] | 3024 | 0.247 | 0.6194 | Yes |
| 67 | NDUFS2 | NADH:ubiquinone oxidoreductase core subunit S2 [Source:HGNC Symbol;Acc:HGNC:7708] | 3072 | 0.243 | 0.6215 | Yes |
| 68 | SDHC | succinate dehydrogenase complex subunit C [Source:HGNC Symbol;Acc:HGNC:10682] | 3145 | 0.235 | 0.6215 | Yes |
| 69 | ATP4B | ATPase H+/K+ transporting subunit beta [Source:HGNC Symbol;Acc:HGNC:820] | 3160 | 0.234 | 0.6260 | Yes |
| 70 | COX7A2 | cytochrome c oxidase subunit 7A2 [Source:HGNC Symbol;Acc:HGNC:2288] | 3164 | 0.234 | 0.6314 | Yes |
| 71 | COX7B | cytochrome c oxidase subunit 7B [Source:HGNC Symbol;Acc:HGNC:2291] | 3452 | 0.207 | 0.6139 | No |
| 72 | PPA2 | inorganic pyrophosphatase 2 [Source:HGNC Symbol;Acc:HGNC:28883] | 3698 | 0.186 | 0.5992 | No |
| 73 | ATP6V1G2 | ATPase H+ transporting V1 subunit G2 [Source:HGNC Symbol;Acc:HGNC:862] | 3709 | 0.184 | 0.6028 | No |
| 74 | COX15 | cytochrome c oxidase assembly homolog COX15 [Source:HGNC Symbol;Acc:HGNC:2263] | 3727 | 0.183 | 0.6058 | No |
| 75 | UQCRH | ubiquinol-cytochrome c reductase hinge protein [Source:HGNC Symbol;Acc:HGNC:12590] | 3827 | 0.174 | 0.6022 | No |
| 76 | ATP6V1F | ATPase H+ transporting V1 subunit F [Source:HGNC Symbol;Acc:HGNC:16832] | 3833 | 0.173 | 0.6060 | No |
| 77 | NDUFA6 | NADH:ubiquinone oxidoreductase subunit A6 [Source:HGNC Symbol;Acc:HGNC:7690] | 3834 | 0.173 | 0.6101 | No |
| 78 | UQCRC2 | ubiquinol-cytochrome c reductase core protein 2 [Source:HGNC Symbol;Acc:HGNC:12586] | 4020 | 0.154 | 0.5993 | No |
| 79 | COX6C | cytochrome c oxidase subunit 6C [Source:HGNC Symbol;Acc:HGNC:2285] | 4036 | 0.152 | 0.6018 | No |
| 80 | SDHD | succinate dehydrogenase complex subunit D [Source:HGNC Symbol;Acc:HGNC:10683] | 4055 | 0.151 | 0.6040 | No |
| 81 | ATP5F1A | ATP synthase F1 subunit alpha [Source:HGNC Symbol;Acc:HGNC:823] | 4137 | 0.143 | 0.6011 | No |
| 82 | ATP6V0A1 | ATPase H+ transporting V0 subunit a1 [Source:HGNC Symbol;Acc:HGNC:865] | 4371 | 0.123 | 0.5858 | No |
| 83 | NDUFAB1 | NADH:ubiquinone oxidoreductase subunit AB1 [Source:HGNC Symbol;Acc:HGNC:7694] | 4445 | 0.118 | 0.5829 | No |
| 84 | COX7C | cytochrome c oxidase subunit 7C [Source:HGNC Symbol;Acc:HGNC:2292] | 4539 | 0.110 | 0.5783 | No |
| 85 | CYC1 | cytochrome c1 [Source:HGNC Symbol;Acc:HGNC:2579] | 4879 | 0.082 | 0.5537 | No |
| 86 | UQCR11 | "ubiquinol-cytochrome c reductase, complex III subunit XI [Source:HGNC Symbol;Acc:HGNC:30862]" | 4917 | 0.078 | 0.5527 | No |
| 87 | UQCRB | ubiquinol-cytochrome c reductase binding protein [Source:HGNC Symbol;Acc:HGNC:12582] | 5003 | 0.073 | 0.5478 | No |
| 88 | COX6A2 | cytochrome c oxidase subunit 6A2 [Source:HGNC Symbol;Acc:HGNC:2279] | 5242 | 0.053 | 0.5305 | No |
| 89 | COX5A | cytochrome c oxidase subunit 5A [Source:HGNC Symbol;Acc:HGNC:2267] | 5329 | 0.045 | 0.5248 | No |
| 90 | UQCRFS1 | "ubiquinol-cytochrome c reductase, Rieske iron-sulfur polypeptide 1 [Source:HGNC Symbol;Acc:HGNC:12587]" | 5509 | 0.032 | 0.5116 | No |
| 91 | ATP6V0A4 | ATPase H+ transporting V0 subunit a4 [Source:HGNC Symbol;Acc:HGNC:866] | 5968 | -0.002 | 0.4759 | No |
| 92 | COX10 | cytochrome c oxidase assembly factor heme A:farnesyltransferase COX10 [Source:HGNC Symbol;Acc:HGNC:2260] | 6092 | -0.012 | 0.4665 | No |
| 93 | ATP5PF | ATP synthase peripheral stalk subunit F6 [Source:HGNC Symbol;Acc:HGNC:847] | 6109 | -0.013 | 0.4656 | No |
| 94 | ATP5ME | ATP synthase membrane subunit e [Source:HGNC Symbol;Acc:HGNC:846] | 6365 | -0.032 | 0.4465 | No |
| 95 | ATP5F1E | ATP synthase F1 subunit epsilon [Source:HGNC Symbol;Acc:HGNC:838] | 6405 | -0.036 | 0.4443 | No |
| 96 | PPA1 | inorganic pyrophosphatase 1 [Source:HGNC Symbol;Acc:HGNC:9226] | 6649 | -0.053 | 0.4265 | No |
| 97 | NDUFA5 | NADH:ubiquinone oxidoreductase subunit A5 [Source:HGNC Symbol;Acc:HGNC:7688] | 6789 | -0.062 | 0.4171 | No |
| 98 | ATP6V1B2 | ATPase H+ transporting V1 subunit B2 [Source:HGNC Symbol;Acc:HGNC:854] | 7074 | -0.086 | 0.3970 | No |
| 99 | TCIRG1 | "T cell immune regulator 1, ATPase H+ transporting V0 subunit a3 [Source:HGNC Symbol;Acc:HGNC:11647]" | 7600 | -0.127 | 0.3590 | No |
| 100 | ATP6V1A | ATPase H+ transporting V1 subunit A [Source:HGNC Symbol;Acc:HGNC:851] | 8013 | -0.156 | 0.3305 | No |
| 101 | ATP6V0A2 | ATPase H+ transporting V0 subunit a2 [Source:HGNC Symbol;Acc:HGNC:18481] | 8843 | -0.221 | 0.2710 | No |
| 102 | ATP4A | ATPase H+/K+ transporting subunit alpha [Source:HGNC Symbol;Acc:HGNC:819] | 10417 | -0.363 | 0.1568 | No |
| 103 | ATP6V1B1 | ATPase H+ transporting V1 subunit B1 [Source:HGNC Symbol;Acc:HGNC:853] | 12206 | -0.646 | 0.0325 | No |
| 104 | MT-ND5 | mitochondrially encoded NADH:ubiquinone oxidoreductase core subunit 5 [Source:HGNC Symbol;Acc:HGNC:7461] | 12743 | -0.906 | 0.0123 | No |
Table: GSEA details [plain text format]

  

Fig 2: KEGG\_OXIDATIVE\_PHOSPHORYLATION      
 Blue-Pink O' Gram in the Space of the Analyzed GeneSet

  

Fig 3: KEGG\_OXIDATIVE\_PHOSPHORYLATION: Random ES distribution      
 Gene set null distribution of ES for **KEGG\_OXIDATIVE\_PHOSPHORYLATION**
